# Metabolism of Photosynthetic Organisms

**DOI:** 10.3390/life11090946

**Published:** 2021-09-10

**Authors:** Tatyana Savchenko, Andrej Frolov

**Affiliations:** 1 Institute of Basic Biological Problems, Pushchino Scientific Center for Biological Research, Russian Academy of Sciences, 142290 Pushchino, Russia; 2 Department of Bioorganic Chemistry, Leibniz-Institute of Plant Biochemistry, Weinberg 3, 06120 Halle (Saale), Germany; 3 Department of Biochemistry, St. Petersburg State University, 199034 St. Petersburg, Russia

According to multiple definitions of life, metabolism is an indispensable characteristic of living organisms. When it comes to autotrophic organisms executing the biosynthesis of organic molecules from the simple inorganic materials, such as water, carbon dioxide, and mineral salts, their metabolism, in essence, implements the transformation of the non-living matter into components of a life form. Photoautotrophic carbon fixation occurring in green plants and photosynthetic bacteria can be considered as the main interface between non-living matter and living organisms, while the complex system of biochemical reactions of central and specialized metabolism enables organisms to maintain growth, development, reproduction, and interaction with the environment.

In this Special Issue of *Life*, we present a collection of articles dedicated to various aspects of research of metabolism in photosynthetic organisms.

Chaudhary and Kalkal [1] present the results of bioinformatics analysis of publicly available transcriptome data generated from the root and shoot tissues of rice genotypes grown under optimal and nitrogen starvation conditions revealing the differentially expressed and alternatively spliced genes. This work extends our understating of nitrogen utilization efficiency mechanisms in the important crop plant.

The occurrence of residual pharmaceuticals in the environment attracted significant attention from the scientific community during the last few decades. In the work of Kudrna et al. [2], lettuce (*Lactuca sativa* L.) plants were employed to probe the effects of one of the most widely used drugs, acetaminophen, on their physiological characteristics. The obtained data demonstrate the high sensitivity of plants to chronic exposure to this omnipresent contaminant.

The manuscript by Mihalik et al. [3] describes the substantial research on isolation and characterization of the specific gene for the production of acetylated triacylglycerols in Euonymus plants. Transgenic host plants overexpressing the diacylglycerol acetyltransferase gene from *E. europaeus* produced and accumulated acetylated triacylglycerols in maturating seeds. Thereby, the difference in fatty acid composition of acetylated triacylglycerols was demonstrated. The presented data provide generalized guidance for the application of the genes from Euonymus plants in biofuel production.

The work by Glabonjat et al. [4] is an important extension of the growing body of knowledge on the biochemistry of arsenosugars and arsenolipids with a special emphasis on their potentially deleterious effects on the organisms representing the basic food web components. The authors present evidence for a trophic transfer of organoarsenicals from the phytoplankton to the zooplankton community and demonstrate the efficient bacterial mineralization of lysis-released organoarsenicals back to inorganic oxyanions before they sink to the sediments. The paper will be of interest to scientists studying the biogeochemical cycling of arsenic.

Comprehensive reviews presented in our Special Issue summarize the recent research advances on the carbon metabolism of purple non-sulfur bacteria, oxidative stress-specific alteration of pant metabolism, and the role of microRNAs in the regulation of plant metabolism and stress tolerance [5,6,7].

Petushkova et al. [5] present a very detailed and comprehensive review summarizing the recent data on the presence and diversity of anaplerotic pathways in purple non-sulfur bacteria. Purple non-sulfur bacteria produce a broad selection of valuable compounds, including molecular hydrogen. These organisms can be cultivated in organic wastewaters, such as fermentation wastes. Thus, these bacteria can be extensively used in biotech applications.

Savchenko and Tikhonov [6] summarize the available data on the alterations in central metabolism associated with plant adaptation to oxidative stress. The analysis provides a theoretical basis for the generation of plants with improved tolerance to oxidative stress and the development of metabolic markers applicable in research and agricultural practice.

The group of authors (Chaudhary et al.) [7] has reviewed the role of microRNAs in plant stress response and their potential use as targets for developing stress-tolerant crops. MicroRNAs mediate post-transcriptional gene silencing and fine-tuning of the regulation of many abiotic and biotic stress-responsive genes in plants. Thus, these molecules might be considered novel targets for engineering stress-tolerant crop varieties.

We believe that the original research and review articles collected in this Special Issue will be interesting to a broad audience of scientists studying physiological, biotechnological, and ecological aspects of autotrophic metabolism.
